# Complete plastome sequence of *Lysimachia congestiflora* Hemsl. a medicinal and ornamental species in Southern China

**DOI:** 10.1080/23802359.2019.1627952

**Published:** 2019-07-11

**Authors:** Hai-Li Li, Xia-Lan Cheng, Yan Chen, Fei-Li Tan

**Affiliations:** Life Science and Technology School, Lingnan Normal University, Zhanjiang, Guangdong, China

**Keywords:** *Lysimachia congestiflora*, plastome, phylogeny, genome structure, Primulaceae

## Abstract

*Lysimachia congestiflora* Hemsl. is a perennial herb of Primulaceae. It is mainly distributed in the provinces to the south of the Changjiang River and south of Shanxi, Gansu province and Taiwan. It is a plant that combines medicinal, ornamental, and economic values. To date no study has been carried out on the genome of *L. congestiflora*. Here, we report and characterize the complete plastid genome sequence of *L. congestiflora* in order to provide genomic resources useful for promoting its conservation. The complete chloroplast genome of *L. congestiflora* is 154,505 bp in length with a typical quadripartite structure, consisting of a large single-copy region (LSC, 84,606 bp), a single-copy region (SSC, 17,961 bp), and a pair of inverted repeats (IRs, 25,969 bp). There are 114 annotated genes, including 80 unique protein-coding genes, 4 unique ribosomal RNA genes, and 30 transfer RNA genes. To investigate the evolution status of *L. congestiflora*, as well as Primulaceae, we constructed a phylogenetic tree with *L. congestiflora* and other 11 species based on their complete chloroplast genomes. According to the phylogenetic topologies, *L. congestiflora* was closely related to *L. coreana.*

*Lysimachia congestiflora* Hemsl. is a plant belonging to the family Primulaceae, mainly distributed in the provinces to the south of the Changjiang River and south of Shanxi, Gansu province and Taiwan. It is a plant that combines medicinal, ornamental, and economic values (Li et al. 2015). The chloroplast genome sequence carries rich information for plant molecular systematics and Barcoding. To date no study has been carried out on the genome of *L. congestiflora.* To provide a rich genetic information and improve *L. congestiflora* molecular breeding in the future, we report and characterize the complete plastid genome sequence of *L. congestiflora* (GenBank accession number: MK834324).

In this study, the fresh leaves of *L. congestiflora* were collected from its natural habitat Gupo Mountain, Hezhou, Guangxi, China (E111°32′23.20″, N24°34′10.15″ E). Voucher specimens (LNH180701015) were deposited in the Herbarium of Lingnan Normal University, Zhanjiang, China. The experiment procedure was as reported in Liu et al. (2016). Total DNA of the *L. congestiflora* was sequenced with second-generation sequencing technology (Illumina HiSeq 2000, San Diego, CA). The chloroplast genome sequence reads were assembled with bioinformatic pipeline including SOAP2 software (Li et al. 2009) and several runs of manual corrections of sequence reads. Genes encoded by this genome were annotated by importing the FASTA format sequence to the DOGMA (Wyman et al. 2004) and recorrected manually. The results showed that plastome of *L. congestiflora* possess a total length 154,505 bp with the typical quadripartite structure of angiosperms, containing two inverted repeats (IRs) of 25,969 bp, a large single-copy (LSC) region of 84,606 bp, and a small single-copy (SSC) region of 17,961 bp. The plastome contains 114 genes, consisting of 80 unique protein-coding genes, 30 unique tRNA genes, and 4 unique rRNA genes. The overall A/T content in the plastome of *L. congestiflora* is 63.00%, for which the corresponding value of the LSC, SSC, and IR regions were 65.20%, 69.60%, and 57.20%, respectively.

We used RAxML (Stamatakis, 2006) with 1000 bootstraps under the GTRGAMMAI substitution model to reconstruct a maximum likelihood (ML) phylogeny of 11 published complete plastomes of Ericales, using *Alniphyllum eberhardtii* and *Changiostyrax dolichocarpus* (Styracaceae) as outgroups. According to the phylogenetic topologies, *L. congestiflora* was closely related to *L. coreana*. Most nodes in the plastome of ML trees were strongly supported (Figure 1). The complete plastome sequence of *L. congestiflora* will provide a useful resource for the conservation of the genetics of this species as well as for the phylogenetic studies for Primulaceae.

**Figure 1.**
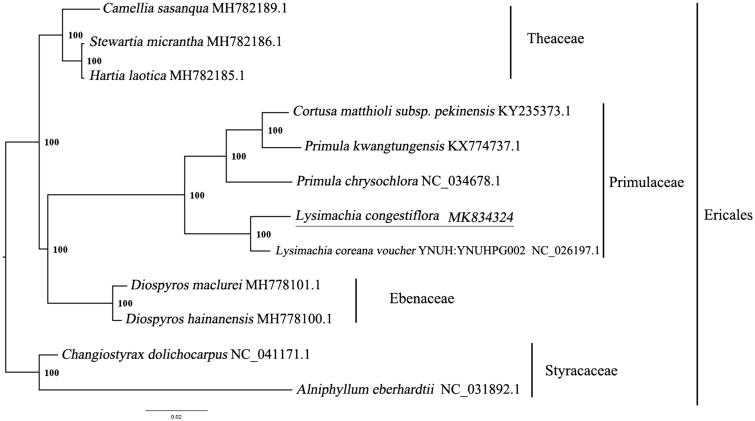
Maximum likelihood phylogenetic tree based on 11 complete chloroplast genomes. Accession number: *Lysimachia congestiflora* (this study); *Camellia sasanqua* MH782189.1; *Stewartia micrantha* MH782186.1; *Hartia laotica* MH782185.1; *Cortusa matthioli* subsp. *pekinensis* KY235373.1; *Primula kwangtungensis* KX774737.1; *Primula chrysochlora* NC_034678.1; *Lysimachia coreana voucher* YNUH:YNUHPG002 NC_026197.1; *Diospyros maclurei* MH778101.1; *Diospyros hainanensis* MH778100.1; outgroup: Changiostyrax dolichocarpus NC_041171.1; Alniphyllum eberhardtii NC_031892.1. The number on each node indicates the bootstrap value.
